# Exome sequencing for paediatric-onset diseases: impact of the extensive involvement of medical geneticists in the diagnostic odyssey

**DOI:** 10.1038/s41525-018-0056-5

**Published:** 2018-08-06

**Authors:** Christopher CY Mak, Gordon KC Leung, Gary TK Mok, Kit San Yeung, Wanling Yang, Cheuk-Wing Fung, Sophelia HS Chan, So-Lun Lee, Ni-Chung Lee, Rolph Pfundt, Yu-Lung Lau, Brian HY Chung

**Affiliations:** 1 Department of Paediatrics & Adolescent Medicine, LKS Faculty of Medicine, The University of Hong Kong, Hong Kong Special Administrative Region, Hong Kong, China; 2 Department of Paediatrics & Adolescent Medicine, Duchess of Kent Children’s Hospital, Hong Kong Special Administrative Region, Hong Kong, China; 3 Department of Pediatrics, National Taiwan University Hospital, Taipei, Taiwan; 4 Department of Human Genetics, Donders Institute, Radboud University Medical Center, Nijmegen, The Netherlands; 5 Department of Obstetrics and Gynaecology, LKS Faculty of Medicine, The University of Hong Kong, Hong Kong Special Administrative Region, Hong Kong, China

## Abstract

Currently, offering whole-exome sequencing (WES) via collaboration with an external laboratory is increasingly common. However, the receipt of a WES report can be merely the beginning of a continuing exploration process rather than the end of the diagnostic odyssey. The laboratory often does not have the information the physician has, and any discrepancies in variant interpretation must be addressed by a medical geneticist. In this study, we performed diagnostic WES of 104 patients with paediatric-onset genetic diseases. The post-exome review of WES reports by the clinical geneticist led to a more comprehensive assessment of variant pathogenicity in 16 cases. The overall diagnostic yield was 41% (*n* = 43). Among these 43 diagnoses, 51% (22/43) of the pathogenic variants were nucleotide changes that have not been previously reported. The time required for the post-exome review of the WES reports varied, and 26% (*n* = 27) of the reports required an extensive amount of time (>3 h) for the geneticist to review. In this predominantly Chinese cohort, we highlight the importance of discrepancies between global and ethnic-specific frequencies of a genetic variant that complicate variant interpretation and the significance of post-exome diagnostic modalities in genetic diagnosis using WES. The challenges faced by geneticists in interpreting WES reports are also discussed.

## Pediatrics: Extra review of exome data boosts diagnostic yield

In-depth reviews by clinical geneticists can improve the diagnostic accuracy of exome sequencing data for children with unexplained genetic disorders, especially in non-Western populations that are under-represented in genomic databases. Working with children predominantly of Han Chinese origin, Brian Chung from the University of Hong Kong and coworkers sequenced the entire protein-coding portion of the genome for 104 patients with pediatric-onset genetic disease. Specially trained geneticists analyzed the DNA data to resolve any ambiguous interpretations, link the molecular findings with clinical records, identify ethnic-specific differences and, when necessary, request additional assays. This extra review process was sometimes laborious, taking several hours of the physician’s time, but ultimately led to a more comprehensive assessment in 16 of the 43 diagnoses successfully made. This overall diagnostic yield—41%—was comparable to previous studies in other populations.

## Introduction

Previous multiple large (*n* > 500) exome projects have reported yields of 25–30%.^1,2^ Subsequent reports have increased the yield to 43%^3^ and even up to 57.7% in selected groups of patients with suspected monogenic disorders.^4^ Most previous studies have been based on data from large centres or consortiums specialising in diagnostic exome sequencing,^1,5,6^ but few studies have focused on the involvement of the medical geneticist in the diagnostic process. Baldridge et al.^3^ published a key study that comprehensively examined the role of the medical geneticist. These authors reported an increase in the diagnostic yield from 36 to 43% after an assessment was performed by a geneticist in a cohort in which minority populations were under-represented. Importantly, the authors found that non-Caucasians had a lower diagnostic yield than Caucasians. This difference was statistically significant even after adjusting for the higher rate of craniofacial anomalies. Thus, we sought to validate this observed low diagnostic yield in non-Caucasians.

The receipt of a WES report can be merely the beginning of a continuing exploration process rather than the end of the diagnostic odyssey. For example, various post-exome diagnostic modalities may be necessary, such as biochemical analyses, imaging studies, and complementary molecular tests. Outside of large institutions specialising in genomics, offering WES via collaboration with an external service laboratory is increasingly common. The laboratory and clinical teams may not be geographically co-located, leading to further challenges. The rapid developments in the clinical use of WES have required medical geneticists to expand their competencies from clinical practice to a variety of new skills, such as 'next-generation phenotyping' for shortlisted exome candidates.^7^ In reality, the expertise required to interpret these findings can no longer be harnessed by a single professional.

The two major aims of this study are, first, to report our findings from an undiagnosed disease programme in a predominantly Chinese population and validate whether the diagnostic yield is indeed low. Second, we demonstrate the role of geneticists in the post-exome review of WES reports, particularly in addressing variants of uncertain significance (VUSs).

## Results

### Patient demographics and sequencing strategy

Among the 104 recruited subjects, 65 subjects were males, and 39 subjects were females; the median age at the time of enrolment was four years and one month (range from one month to 33 years). In total, WES of 81 patients (78%) were analysed as singletons, and 23 patients (22%) were analysed as trios. Seventy-nine patients (76%) were offered WES fully supported by study funding, and the remaining patients paid either fully (*n* = 16) or partially (*n* = 9) out of pocket, i.e., the parents self-financed their own exomes, while the cost of sequencing of the proband was supported. The overall average turnaround time for both laboratories to provide the initial report was 20 weeks (range from 3–39 weeks).

### Indications for testing

The clinical presentations were classified according to the primary indication for testing. A standard laboratory check list was used for the exome requests, and the primary panel of genes analysed was based on this indication. The most common indication for testing was neurological disorders (67%), including intellectual disabilities (*n* = 34), movement disorders (*n* = 12), neuromuscular diseases (*n* = 11), mitochondrial diseases (*n* = 5) and epilepsy (*n* = 8). The remaining indications included multiple congenital anomalies (*n* = 17, 16%) or other suspected genetic disorders (*n* = 17, 16%) (Supplementary Figure 1).

### Diagnostic yield

**Fig. 1:**
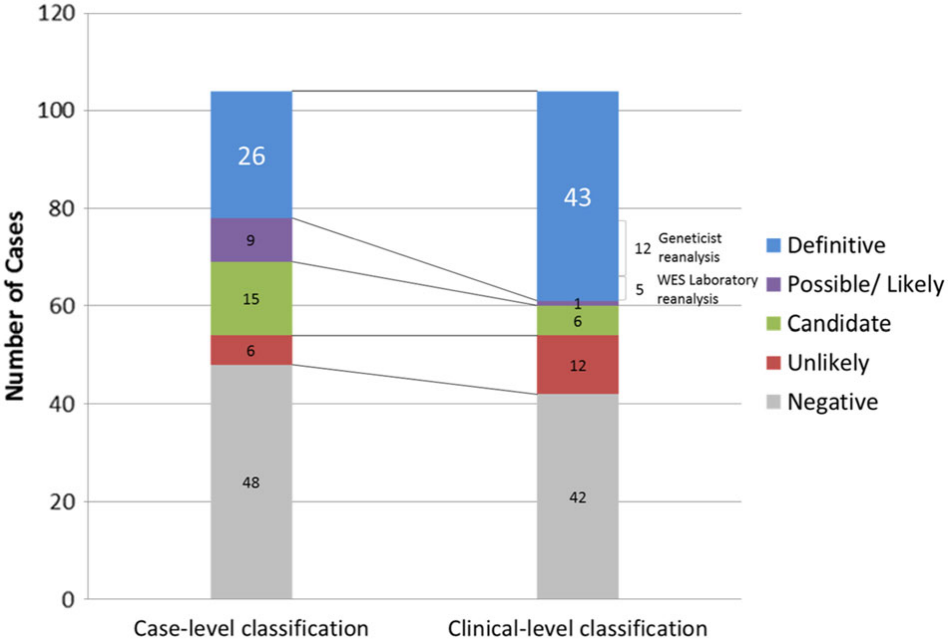
Change in classification after reanalysis. The number of definitive diagnoses increased from n = 26 to n = 43. This was a result of both laboratory (n = 5) and clinician (n = 12) initiated reanalysis. The number of both “negative” (n = 6) and “*candidate*” variants of uncertain significance (n = 9) was reduced. Therefore increasing the yield and reducing ambiguity

Immediately after the targeted parental Sanger sequencing of the singleton exomes, among all 104 cases, 26 cases (25%) were classified as definitive (Category 1) diagnoses, and a clear molecular diagnosis was established. The reanalysis by the geneticist increased this yield by 12 cases, including 10 cases that were promoted to definitive (Category 1), and two additional variants were discovered (*FGD1* and *IGHMBP2*) (Table 1). During the duration of this project, the laboratory subsequently reanalysed the WES data due to updated bioinformatics pipelines^8^ or newly published studies, and five additional diagnoses were revealed in a supplementary report. These diagnoses included pathogenic variants in *PPP1CB*,^9^ *DDX3X*^10^ and *ASXL3*^11^ and two copy-number variations, i.e. an 8-Mb loss of 10q26.2-qter and a mosaic 33.89-Mb gain of 12p13.33-p11.1 (Pallister–Killian syndrome). Overall, a conclusive diagnosis was achieved in 43 cases (41%) (Fig. 1). Excluding the two CNV diagnoses, of the 41 cases, 21 autosomal dominant (AD), 12 autosomal recessive (AR) and eight X-linked (XL) diseases were diagnosed. Of these diseases, 24 (59%) cases were de novo mutations. Overall, 22 reports (51%) involved novel mutations that have not been previously reported (literature search was performed in May 2017). Three of these novel missense variants were found in a codon that was also the site of a different variant that had previously been reported as pathogenic. These diagnoses included more well-known disease genes (e.g. *HRAS, ATRX, CREBBP, NTRK1, FANCA, UPB1, SBDS, SPG11, STXBP1, AGRN, GRIN2A, CHKB* and *PACS1*) and genes that have only been reported recently (e.g. *PURA, DDX3X, WAC, PPP1CB* and *KMT2B*), as shown in Table 2. All variants identified in this study are summarised in Supplementary Table 1.

**Table 1:** Influence of post-exome review of WES reports by clinical geneticist

ID	Sex	Singleton or Trio	Gene	Variants reclassified/ discovered	Inheritance	Case-level classification	Clinical-level classification	Change in classification	Description	I. Post-exome phenotyping	II. Post-exome diagnostics	III. Extensive database evaluation	IV. Expert liaison	V. Clinical functional assay
U023	F	Singleton	*UPB1*	c.977G>A(p.Arg326Gln) Hom	AR (Inherited)	Candidate	Definitive	Promoted	Initially thought to be a VUS due to relatively high MAF in East Asians. The variant has been subsequently reported multiple times in the Chinese and Japanese populations. Discussion with experts and testing urine for purines and pyrimidines confirmed the diagnosis.		●	●	●	
U024	F	Singleton	*MECP2*	c.1126C>T (p.Pro376Ser) Het	AD (inherited)	Possible	Unlikely	Demoted	First reported to be a possible cause, review of Rett disease-specific database showed that this was more likely a common benign polymorphism.			●		
U027	M	Singleton	*SBDS*	c.184A>T(p.Lys62*); c.258+2T>C (r.spl?) Het	AR (Inherited)	Candidate	Definitive	Promoted	A VUS in the *GRIN2A* gene was initially ruled out after discussion with experts. This was communicated back to the laboratory and further mutations in SBDS were revealed. These were not included in the initial report as WES was unable to separate the normal gene from pseudogene with 97% homology. By obtaining specific primers from experts, the diagnosis was confirmed.		●		●	
U028	M	Singleton	*COQ4*	c.402+1G>A Het; c.550T>C (p.Trp184Arg) Het	AR (Inherited)	Possible	Definitive	Promoted	Functional testing for *COQ4* enzyme essay was arranged to confirm the pathogenicity of a VUS (c.550T>C).				●	●
U029	M	Singleton	*SPAST*	c.1045T>G (p.Leu349Val) Het c.1824C>G (p.Asn608Lys) Het	AR (Inherited)	Candidate	Unlikely	Demoted	Further clinical samples from family members and consultation of expert groups demoted this to a likely benign variant.		●		●	
U031	F	Singleton	*PIGO*	c.2191del (p.Arg731fs) Het c.458T>C (p.Phe153Ser) Het	AR (Inherited)	Possible	Definitive	Promoted	Atypical presentation of this patient with normal Alkaline phosphatase required expert liaison and further functional studies by flow cytometry to confirm the diagnosis.		●		●	●
U033	F	Singleton	*SLC35A2*	c.569dup (p.Gly191fs) Het	XL (de novo)	Possible	Definitive	Promoted	Consultation of expert groups, X-inactivation studies and transferrin electrophoresis was arranged to confirm the pathogenicity of this previously unreported variant.		●		●	
U039	F	Singleton	*NF1*	c.2970_2972del (p.Met992del) Het	AD (de novo)	Possible	Definitive	Additional Finding	Although the patient phenotype was consistent with a variant in *CREBBP*, the finding of café au lait spots on examination prompted reanalysis for an additional *NF1* variant.	●				
U049	M	Singleton	*ATP6V1A*	c.215G>A (p.Gly72Asp) Hom	AR (inherited)	Candidate	Definitive	Promoted	Collaboration with expert groups allowed complex functional studies and discovery of this new disease gene *ATP6V1A*.		●		●	●
U051	M	Singleton	*COL6A2*	c.1358G>A (p.Arg453His) c.1706G>A (p.Arg569Gln) c.3003C>A (p.Asp1001Glu)	AD/AR (n/a)	Candidate	Unlikely	Demoted	Consultation of an international expert confirmed that the candidate gene did not fit with the known phenotype.				●	
U068	M	Singleton	*CSNK2A1*	c.593A>G (p.Lys198Arg)	AD (de novo)	Candidate	Definitive	Promoted	On initial report, this variant has not been reported before, but several other similar cases were identified by the WES laboratory. Collaboration to aggregate cases resulted in a publication (Chiu et al. 2018).			●	●	
U070	M	Singleton	*GJB2*	c.109G>A (p.Val37Ile) Hom	AR (inherited)	Definitive	Unlikely	Demoted	Discussion with various groups demoted this variant of variable expressivity and penetrance to a VUS.				●	
U096	F	Singleton	*ABCB11*	c.3429C>G (p.Asp1143Glu) c.2594C>T (p.Ala865Val)	AR (inherited)	Possible	Unlikely	Demoted	Phenotype correlation and review of local databases demoted this to an unlikely diagnosis.	●		●		
U098	M	Singleton	*IGHMBP2*	c.2356del (p.Ala786fs) c.905_912+83del	AR (inherited)	Candidate	Definitive	Additional Finding	Liaison with laboratory facilitated reanalysis of exome data and discovery of an additional 83 bp deletion in an AR disease.	●				
U003	M	Trios	*ATRX*	c.740A>G (p.Asn247Ser)	XL (inherited)	Possible	Definitive	Promoted	Atypical presentation at 4 months old with no suggestion of alph-thalassemia or intellectual disability, review and follow-up of patient found low MCV on CBP and developmental delay, arranging a test for Hb H inclusion bodies confirmed the diagnosis.		●			
U030	M	Trios	*ARID1A*	c.3406G>A (p.Ala1136Thr)	AD (de novo)	Candidate	Definitive	Promoted	Originally reported as a novel variant of unknown significance. Further review of the phenotype and literature was required to confirm the diagnosis.	●		●		
U103	M	Trios	FGD1	exon 5 deletion	XL (inherited)	Negative	Definitive	Additional Finding	Exome report was initially negative i.e. no candidate variants identified. Expert opinion suggested targeted analysis of genes for Robinow syndrome and Aarskog–Scott syndrome was performed, for which no mutations were found on initial WES report. Further discussion with the exome laboratory identified low coverage areas in the FGD1 gene, and a deletion was identified by MLPA.	●			●	
										**5**	**7**	**5**	**11**	**3**

Post-exome review of WES reports led to a more comprehensive assessment in 16 cases. One additional finding (U103) was discovered by reanalysis of a negative report

**Table 2:** All 43 positive diagnoses and management categories

ID	Sex	Age (year/months)	Diagnosis (OMIM)	Gene	Management Category	R	D	P	S	L	M	O
U001	F	1 yr/9 mo	Mental retardation, X-linked 102 (OMIM #300958)	*DDX3X*	Category 3							
U003	M	1 yr/1 mo	Alpha-thalassemia/mental retardation syndrome, X-linked (OMIM #301040)	*ATRX*	Category 1	●	●			●	●	
U004	F	6 yr/4 mo	Epilepsy, focal, with speech disorder with or without mental retardation (OMIM #245570)	*GRIN2A*	Category 2		●					
U005	M	0 yr/11 mo	Fanconi anaemia, complementation group A (OMIM #227650)	*FANCA*	Category 1		●	●	●		●	
U006	M	1 yr/11 mo	Schuss–Hoeijmakers sydnrome (OMIM #615009)	*PACS1*	Category 1	●	●			●		
U010	F	9 yr/9 mo	Axenfeld–Rieger syndrome, type 3 (OMIM #602482)	*FOXC1*	Category 3				●			
U012	F	2 yr/4 mo	10q26 microdeletion syndrome (OMIM #609623)	10q26.2-qter deletion	Category 2		●					
U015	M	11 yr/10 mo	Coffin-Siris syndrome 1 (OMIM #135900)	*ARID1B*	Category 1	●	●		●			
U017	M	33 yr/3 mo	Autosomal recessive; Spastic paraplegia 11, autosomal recessive (OMIM #604360)	*SPG11*	Category 2	●						
U023	F	2 yr/11 mo	Beta-ueridopropionase deficiency (OMIM #613161)	*UPB1*	Category 2		●					
U025	F	20 yr/1 mo	Bainbridge–Ropers syndrome (OMIM# 615485)	*ASXL3*	Category 2	●					●	
U027	M	1 yr/2 mo	Shwachman–Bodian–Diamond syndrome (OMIM #260400)	*SBDS*	Category 1	●	●	●	●		●	
U028	M	2 yr/6 mo	Coenzyme Q10 deficiency, primary, 7 (OMIM #616276)	*COQ4*	Category 2						●	
U030	M	1 yr/3 mo	Coffin-Siris syndrome 2 (OMIM # 614607)	*ARID1A*	Category 1	●	●		●			
U031	F	11 yr/8 mo	Hyperphosphatasia with mental retardation syndrome (HMRS) 2 (OMIM #614730)	*PIGO*	Category 2		●					
U033	F	5 yr/0 mo	X-linked congenital disorder of glycosylation type Iim (OMIM #300896)	*SLC35A2*	Category 3							
U039	F	7 yr/11 mo	Rubinstein–Taybi syndroom type 1 (OMIM #180849)	*CREBBP*	Category 1	●			●			
U040	F	8 yr/3 mo	Bainbridge–Ropers syndrome (OMIM# 615485)	*ASXL3*	Category 2	●					●	
U042	F	13 yr/3 mo	Early Infantile Epileptic Encephalopathy (OMIM #612164)	*STXBP1*	Category 1				●		●	
U044	M	0 yr/1 mo	Noonan syndrome-like disorder with loose anagen hair 2 (OMIM #617506)	*PPP1CB*	Category 1	●	●		●			
U049	M	0 yr/1 mo	Cutis laxa, autosomal recessive, type IID (OMIM #617403)	*ATP6V1A*	Category 3							●
U050	M	4 yr/0 mo	Lenz–Majewski hyperostotic dwarfism (LMHD) (OMIM #151050)	*PTDSS1*	Category 2	●	●		●			
U052	M	4 yr/3 mo	Autosomal recessive agenesis of the corpus callosum with peripheral neuropathy (OMIM #218000)	*SLC12A6*	Category 3							
U055	M	5 yr/10 mo	Wieacker–Wolff syndrome, X-linked recessive (OMIM #314580)	*ZC4H2*	Category 3							
U062	F	5 yr/4 mo	Neurodevelopmental disorder a.o. Rett syndrome (OMIM #312750)	*MECP2*	Category 1	●			●		●	
U063	M	8 yr/6 mo	Congenital megaconial muscular dystrophy (OMIM #602541)	*CHKB*	Category 2				●	●	●	
U068	M	10 yr/8 mo	Okur–Chung neurodevelopmental syndrome (OMIM #617062)	*CSNK2A1*	Category 3							
U069	M	4 yr/1 mo	Mental retardation, autosomal dominant 31 (OMIM #616158)	*PURA*	Category 3	●			●			
U074	M	0 yr/8 mo	Pallister–Killian syndrome (OMIM #601803)	12p13.33-p11.1 duplication	Category 2	●			●			
U076	M	1 yr/3 mo	Keratitis-ichthyosis-deafness syndrome (OMIM #148210)	*GJB2*	Category 2	●			●	●		
U080	F	3 yr/8 mo	Mental retardation, X-linked 102 (OMIM #300958)	*DDX3×*	Category 3							
U081	M	4 yr/1 mo	Desanto–Shinawi syndrome (OMIM #616708)	*WAC*	Category 2				●			
U083	F	8 yr/5 mo	X-linked dominant Neurodegeneration with Brain Iron Accumulation 5, NBIA5 (OMIM #300894)	*WDR45*	Category 2		●		●			
U084	F	16 yr/11 mo	Spinal muscular atrophy, lower extremity-predominant 1, AD (OMIM #158600)	*DYNC1H1*	Category 2		●		●			
U087	F	6 yr/10 mo	Auriculocondylar syndrome 1 (OMIM #602483)	*GNAI3*	Category 2	●						
U089	F	17 yr/5 mo	Hereditary sensory and autonomic neuropathy type IV (HSAN IV)	*NTRK1*	Category 2				●	●		
U090	M	4 yr/2 mo	Costello syndrome (OMIM #218040)	*HRAS*	Category 1	●	●		●		●	
U092	F	2 yr/6 mo	Myasthenic syndrome, congenital, 8, with pre- and postsynaptic defects (OMIM #615120)	*AGRN*	Category 2	●			●		●	
U098	M	6 yr/6 mo	Charcot–Marie–Tooth disease, axonal, type 2S (OMIM #616155)	*IGHMBP2*	Category 2					●		
U099	M	1 yr/4 mo	Nicolaides–Baraitser syndrome (OMIM #601358)	*SMARCA2*	Category 1				●			
U100	M	6 yr/0 mo	Mental retardation, autosomal dominant 7 (OMIM #614104)	*DYRK1A*	Category 1				●			
U102	M	9 yr/9 mo	Dystonia 28, childhood-onset (OMIM #617284)	*KMT2B*	Category 2	●		●			●	
U103	M	0 yr/4 mo	Aarskog–Scott syndrome (OMIM #305400)	*FGD1*	Category 3		●		●			
					Total	19	16	3	23	6	12	1

Details of each management category are given in Supplementary Table 2

*R* Referral, *D* Diagnostic testing, *P* Procedure, *S* Surveillance, *M* Medication, *L* Lifestyle changes, *O* Others

### Number of cases requiring an extensive analysis

We calculated the time spent on the post-exome analysis as described in the Methods. In total, 40 cases were negative reports, which required no further input from the geneticist. Of the remaining reports, 27 of the 104 (26%) cases required more than 3 h to review, reflecting the extensive workload. This in-depth review by the clinical geneticist was particularly helpful in several ways: (1) detailed review of conflicting interpretations of pathogenicity in literature, (2) comparison of global and ethnic-specific allele frequencies, (3) request of additional clinical functional assays, and (4) correlation of molecular findings with the clinical context.

### Management implications of WES

**Fig. 2:**
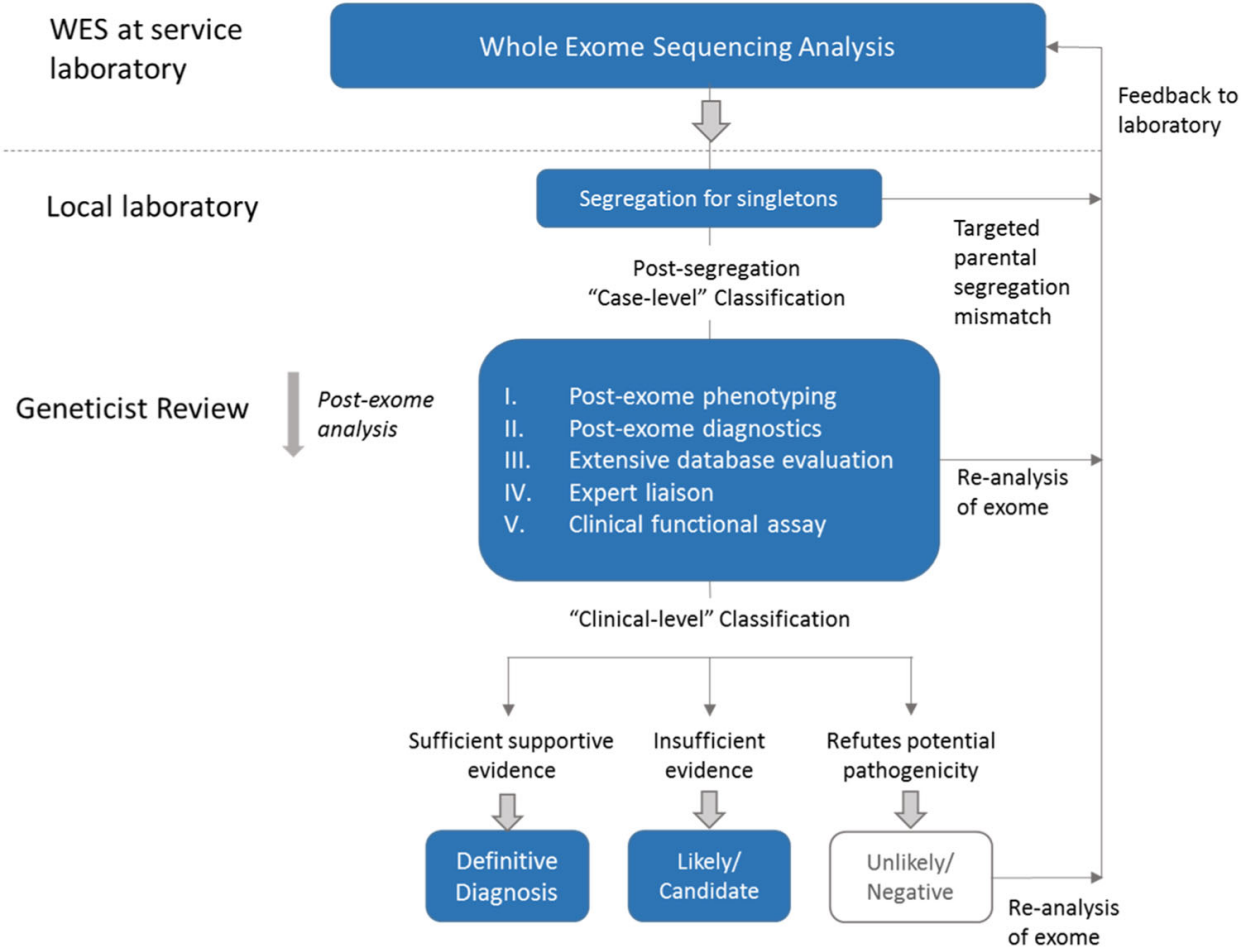
Strategy for geneticist review of exome sequencing reports. An overview of the analysis process by the geneticist. Singleton results first undergo targeted parental segregation to elicit the mode of inheritance. After this, a ‘post-exome analysis’ is performed by the medical geneticist, consisting of 5 key areas of actions. Subsequent interpretation of variants may be fed back to the laboratory at any stage of the process

Among all 43 positive diagnoses, 77% (*n* = 33) of the WES diagnoses provided further information aiding clinical management. This management could be based on disease-specific published management guidelines, i.e. Category 1 (*n* = 13, 30%), or case reports or the known function of genes, i.e. Category 2 (*n* = 20, 47%) (Supplementary. Figure 2), utilising the classification adopted by Stavropoulos et al.^12^ Six major types of interventions were classified according to Riggs et al.^13^ We observed that WES most commonly aided management in the areas of surveillance (53%) and specialist referral (44%) (Table 2 and Supplementary Table 2). However, the implications on clinical outcomes are difficult to measure without an extensive longitudinal follow-up. The details of the management changes are as follows:

i. A referral to a specialist was made in 19/43 (44%) cases after the WES diagnosis. For example, a diagnosis of Lenz–Majewski hyperostotic dwarfism (LMHD) prompted referrals to endocrinology for a DEXA scan and orthopaedics for a skeletal assessment (U050).
ii. Further diagnostic testing was ordered in 16/43 (37%) cases. In a patient (case U027) diagnosed with Shwachman–Bodian–Diamond syndrome, exocrine pancreatic dysfunction is a known anomaly in this condition, and by ordering abdomen CT, evidence of pancreatic lipomatosis was found. In another case (U084), the patient was diagnosed with lower extremity-predominant spinal muscular atrophy. Thus, an MRI of the leg muscle was ordered to further elucidate the diagnosis and severity.
iii. Surgical or interventional procedures were indicated or contraindicated after a genetic diagnosis was achieved in 3/43 (7%) cases. In a patient with early-onset generalised dystonia (U102), a mutation in *KMT2B* causing childhood-onset dystonia was identified, leading to multiple benefits for counselling and management. First, the life expectancy was reportedly extended into adulthood. Furthermore, dopamine treatment for Parkinsonism was shown to be ineffective,^14^ and the patient was referred for deep brain stimulation after performing a literature review. In a case of infantile Fanconi anaemia (complementation group A), the genetic diagnosis led the paediatrician to arrange for matched-sibling cord blood transplantation for the child at the age of 13 months.
iv. Surveillance for potential complication(s) associated with genetic variants was advised in 23/43 (53%) case. This recommendation was the most common among all changes in clinical management. For example, regular assessments for developmental, ophthalmic, and intracranial manifestations were advised for children with *NF1* mutations.^15^
v. Medication was indicated or contraindicated in 12/43 (28%) patients. For example, a mutation in *ARGN* was identified in a patient refractory to treatment with acetylcholinesterase inhibitors for congenital myasthenia gravis (U092). The option of treatment with salbutamol was prompted after a review of the latest literature.^16^ The genetic diagnosis may also flag new contraindications for medications. In congenital megaconial muscular dystrophy associated with *CHKB* variants, reports have suggested the possibility that inter-current vaccinations or anaesthesia can lead to deterioration in muscle or cardiac function; thus, extra caution is advised.^17^
vi. Lifestyle changes were advised in 6/43 (14%) cases, including sun protection against the potential complication of squamous cell carcinoma in a patient with a mutation in *GJB2* or keratitis-ichthyosis-deafness syndrome.

## Discussion

The key aims of this study are to report our findings in a non-Caucasian predominantly Han Chinese population (92%) and to examine the role of medical geneticists in the diagnostic odyssey of WES, particularly in the post-exome analysis of laboratory variant reports.

In this cohort of 104 patients, the overall diagnostic yield was 41% (*n* = 43), which is consistent with the previously reported range of 25–57.7%.^1–4^ The Han Chinese population has been under-represented in WES studies, but this population constitutes the largest ethnic group worldwide.^18^ We provide further data from this ethnic group to expand the spectrum of molecular diagnoses achieved and to compare the diagnostic yield. Although a direct comparison of diagnostic yield is difficult, particularly due to variations in the selection criteria, our findings show that WES can be utilised in the Han Chinese population with a yield comparable to that reported in major studies.

### Areas of post-exome analysis affecting outcome

Five key areas of the post-exome analysis led to a different clinical-level classification of the variants from the case-level classification derived from the laboratory data.

#### Post-exome phenotyping

Post-exome phenotyping can often be helpful in reaching a more complete diagnosis, particularly when the neurological profile of paediatric patients changes over time or the specific phenotypic features do not fit the proposed molecular diagnosis, e.g. a blended phenotype. For example, in a patient diagnosed with Rubinstein–Taybi syndrome, a careful examination revealed the presence of café au lait spots in both the child and father. This suspicion led to a targeted reanalysis and the discovery of a second genetic diagnosis involving the *NF1* gene (U039). In such cases, the geneticist’s proactive effort to review the WES findings and effective communication with the laboratory can facilitate a more comprehensive diagnosis.

#### Post-exome diagnostics

Various inherited diseases inevitably require additional diagnostics to further confirm the diagnosis. These diagnostic assessments include additional clinical biochemical and radiological tests, molecular diagnostic modalities, e.g. Multiplex Ligation-dependent Probe Amplification (MLPA), and segregation of the variant allele within the extended family.

Examples of further clinical testing include (1) purines and pyrimidines for beta-ureidopropionase deficiency (U023), (2) X-inactivation studies and transferrin electrophoresis for X-linked congenital disorder of glycosylation type II (U033), and (3) Hb H inclusion bodies for alpha-thalassemia/mental retardation syndrome (U003).

An additional example of molecular diagnostic modalities includes a single variant in *IGHMBP2*, which was reported in a patient with suspected autosomal recessive Charcot–Marie–Tooth disease. Due to a strong suspicion based on the phenotypic features, the geneticist prompted the laboratory to search for other variants in the gene. A second 83 bp deletion was confirmed by Sanger sequencing (U098) after the exome data were deemed suspicious.

In a few cases, segregation data of extended family members may also help provide further evidence, particularly in families with suspected AR diseases and multiple siblings.^19^ As described by the ACMG guidelines for variant interpretation,^20^ the testing of extended family members can strengthen or refute the pathogenicity of the variants, such as in cases U029 (*SPAST*), U031 (*PIGO*), and U049 (*ATP6V1A*). This process is also best initiated by the medical geneticist

#### Extensive database evaluation

The bulk of the time spent by the geneticist involved an extensive review of the latest studies and databases to identify novel variants.

In Hong Kong, the assessment of ethnic-specific variants has been a challenge due to the lack of a large local variant database. This situation has improved with the recent availability of ethnic-specific exome aggregation data. For example, the homozygous variant *GJB2:*p.(V37I) was initially classified as likely pathogenic based on a WES report (U070). However, a manual search of the variant in gnomAD identified 80 homozygous alleles exclusively in East Asians. After discussion with the WES laboratory and various experts, this variant was considered likely a hypomorphic allele with a partial effect on hearing loss.

Another, more complicated example is the *UPB1* (p.(R326Q)) variant. Despite the minor allele frequency of 2.5% in East Asians, this variant has been reported multiple times as pathogenic in Chinese and Japanese populations.^21–23^ In particular, Nakajima et al.^21^ identified eight homozygotes among affected patients, which was supported by functional studies showing a dramatically reduced residual activity of the mutant βUP. We arranged for a further urine test to measure the pyrimidine degradation metabolites in our patient, and the pyrimidines were highly elevated, e.g. the N-carbamyl-ß-amino acids were elevated from the baseline of 0.50 to 16.65 (over a 30-fold increase). The neurologist confirmed that the phenotypic features of cerebella atrophy and seizures were consistent with the diagnosis. This patient was further discussed with an expert in inborn errors of purine and pyrimidine metabolism, who also confirmed the pathogenicity of this variant.

#### Expert liaison

The process of involving other experts overseas or discussing cases with specialists in the field had the greatest impact on the five key areas, and most cases in which the interpretation was altered required discussion with relevant experts (11/16, 69%). In the case of uncertainty, expert liaison is often an effective strategy for clarifying the overall interpretation and filling in the gaps in knowledge.

For example, in case U103, the exome was negative, and the phenotype and photographs were reviewed with an expert in dysmorphology. This review raised the suspicion of Aarskog–Scott syndrome, and a molecular diagnosis was identified by a targeted reanalysis of the *FGD1* gene (U103). Similarly, many cases involved experts in a relevant field because the experts are more proficient at recognising relevant phenotypes or mutational hot spots. For example, case U051 was a patient with myopathy, and a collagen disorder was suspected; three VUSs were identified in the *COL6A2* gene. The involvement of an expert enabled a comprehensive assessment of the clinical features, muscle biopsy results and molecular findings. It was determined that the presentation did not fit known cases of *COL6A2* myopathies, demoting this to an unlikely genetic diagnosis.

#### Clinical functional assays

Functional tests are helpful in confirming the pathogenicity of variants and can be performed through collaboration with other laboratories. Examples include (i) flow cytometry of patient granulocytes demonstrating GPI-anchor deficiency^24^ for these novel variants in the *PIGO* gene (U032) and (ii) reduced complex II + III activity in fibroblasts in a patient with novel *COQ4* variants (U028). The collaboration in both cases was crucial for confirming these novel variants as pathogenic variants.

### Cases requiring extensive time to review

The time required to review these reports greatly varied. We highlight that 26% (*n* = 27) of all reports required an extensive amount of time (>3 h) to review. Most cases were novel variants which can be attributed to the fact that 24 diagnoses were in genes discovered after the project was started in 2012. These genes included *ATP6V1A, PPP1CB, KMT2B, DDX3X*, *COQ4, WAC, PTDSS1, PURA, IGHMBP2, ASXL3, SLC35A2, ZC4H2, PACS1, ARID1B, ARID1A, PIGO, WDR45, DYNC1H1, GNAI3, SMARCA2*, and *DYRK1A*. More importantly, 21 diagnoses (49%) of novel mutations have never been previously reported (Supplementary Table 1).

The abundance of newly described diseases and previously unreported variants highlight the power of WES as a diagnostic tool, but, simultaneously, extensive involvement by a geneticist is required to reaffirm these findings. Therefore, geneticists and non-specialists should be particularly aware of these scenarios because more input from experts in genetics is likely required. In more resourceful centres, the ideal model involves a multidisciplinary team to examine and interpret these variants. However, in a setting where genetic expertise is limited, pursuing these cases may not be sustainable in the long term, and additional staff is required to ensure adequate service delivery. This excessive workload will likely be exaggerated when the option of WES is opened to non-genetic specialists, and an accurate and comprehensive interpretation of novel variants greatly depends on the genetic expertise of the clinical team.

The laboratory often does not have the information the physician has, and it is not a simple matter of performing a molecular analysis at the laboratory and then following up on the variant in the clinical setting. This challenge is particularly apparent when the laboratory and clinical teams are not geographically co-located. This geographical separation results in cross-talk by electronic communication, and the clinical team must often perform an extensive analysis of the novel VUSs. In cases of difficult novel variants, the clinical team can simply choose to accept these as VUSs, but the proactive effort to review these variants can have a substantial impact, even if it only results in 12% more diagnoses (12/104).

### Study limitations

The limitations of this study include the relatively small cohort size (*n* = 104), but this sample provides a good representation of the load of WES cases observed over the period of four years. Notably, the singleton exome strategy was used in 78% of the cohort. The choice of a singleton strategy was due to financial constraints, and 88 of the 104 cases were supported by the study funding. Certain data suggest that singleton exome sequencing as a first-tier test outperforms standard care^4^; thus, we adopted this at our clinic. While this practice can be a limitation to our study, many laboratories accept singleton exomes. We found this practice to be a feasible alternative for patients who cannot pay out of pocket for the cost, but additional resources for the segregation analysis are required. Another limitation is the use of two different laboratories and the inclusion of six additional patients from Taiwan. In practice, referrals consist of patients not only from Hong Kong but also from mainland China and other parts of Southeast Asia. Thus, we did not exclude the Taiwanese patients from our selection of patients, and we believe that our sample is a realistic representation of Han Chinese patients seeking genetic diagnoses in the Asia Pacific region.

## Conclusion

In our predominantly Han Chinese cohort, the overall diagnostic yield of WES was 41%. This is comparable to that observed in previous studies involving Caucasian populations. We found that in-depth review by the clinical geneticist was most helpful in several areas: (1) detailed review of conflicting interpretations of pathogenicity in literature, (2) comparison of global and ethnic-specific allele frequencies, (3) request of additional clinical functional assays, and (4) correlation of molecular findings with the clinical context. Clinical geneticists will likely make the most impact by focusing on these aspects to improve the overall interpretation of WES reports. For the same reasons, we found that 26% (*n* = 27) of WES reports required an extensive amount of time (>3 h) for the geneticist to review. Therefore, clinical genetics services should be aware of the additional workload created and the extra resources required to deal with the demands created by the use of WES.

## Materials And Methods

### Patient recruitment

The Department of Paediatrics and Adolescent Medicine at the Queen Mary Hospital is a tertiary referral centre affiliated with The University of Hong Kong. The service includes the neighbouring Duchess of Kent Children’s Hospital, which specializes in developmental paediatrics, rehabilitation and paediatric neurology. Both in-patient and out-patient clinical genetic consultations are available at these two hospitals. Most patients are residents of Hong Kong or mainland China. In addition, six patients from National Taiwan University Hospital were included in our cohort. Overall, 94% of the patients were of self-reported Han Chinese ethnicity.

Patients were prospectively recruited from November 2012 to November 2016 by referrals to the clinical genetics service for undetermined diagnoses. The indications included neurological disorders, multiple congenital anomalies and other clinical presentations with a strong suspicion of a monogenic cause. WES was offered to patients meeting the indications for testing outlined by ACMG.^25^ A medical geneticist assessed all patients for inclusion, and patients with a clearly recognisable genetic syndrome or condition were excluded from this study (targeted genetic tests were ordered for these patients). Chromosomal microarray was performed for all patients referred within the same hospital. This was also strongly recommended for referrals from other hospitals.

Notably, WES is not currently provided by any molecular laboratory in the public health care system in Hong Kong during the study period; thus, the samples were sent to laboratories overseas. All families were first offered a self-financed trio WES at the clinic. When the families could not afford this out of pocket expense, a singleton WES of the proband was offered and supported by the study funding. This strategy of singleton WES was chosen to maximise the utility of the limited funding resources.

### Whole-exome sequencing

The exome sequencing was performed by two laboratories. Of all reports, 95 reports were analysed by Genome Diagnostics Nijmegen (Nijmegen, Netherlands), and nine reports were analysed by Ambry Genetics (Aliso Viejo, CA).

For the WES performed at Genome Diagnostics Nijmegen, the targets were enriched using Agilent SureSelectXT (Agilent Technologies), and the whole-exome sequencing was performed on an Illumina HiSeq platform (BGI, Copenhagen, Denmark), followed by data processing using BWA (read alignment) and GATK (variant calling). The variants were annotated using the external laboratory’s in-house-developed pipeline. The variants were prioritised using an in-house-designed ‘variant interface’ and manual curation.^13^ For the WES performed at Ambry Genetics, the samples were prepared using a SureSelect Target Enrichment System (Agilent Technologies) or SeqCap EZ VCRome 2.0 (Roche NimbleGen) and sequenced on an Illumina HiSeq 2000 or 2500. The initial data processing, base calling, alignments and variant calls were performed using various bioinformatics tools at Ambry Genetics. The variant calls were annotated using the Ambry Variant Analyzer tool (AVA)^26^ and filtered using laboratory-devised strategies.

### Post-exome review by a medical geneticist

All WES reports received from the overseas laboratories were re-evaluated by a medical geneticist regardless of the variant classification (Fig. 2). The steps can be summarised into the following five main categories: (I) post-exome phenotyping of any changes in the phenotype or additional features, i.e. blended phenotype; (II) post-exome diagnostics, such as additional molecular tests, segregation studies of extended family members or clinical biochemical and radiological investigations; (III) extensive database evaluations, including ClinVar, HGMD, disease-specific databases, and population frequency databases of ethnic-specific allele frequencies, e.g. ExAC (http://exac.broadinstitute.org) or Taiwan biobank (https://taiwanview.twbiobank.org.tw/); (IV) expert liaison for opinions regarding potential diagnoses and collaboration for aggregation of cases; and (V) functional experimental studies that can only be performed at specialised research laboratories. If further work on the WES data was required after this review process, the information was communicated to the laboratory.

### Case-level and clinical-level classifications

Baldridge et al.^3^ utilised the following three levels of variant classifications: (1) variant level, (2) case level and (3) clinical level. A variant-level assertion is a raw interpretation of the variant, whereas a case-level assertion is a synthesis of all molecular data regarding a single subject by the laboratory. Using the same definition, the WES reports from Ambry and Nijmegen in this study did not clearly distinguish between the two. In most reports from Nijmegen, the terms used to describe the likelihood of pathogenicity included ‘probably the cause’ or ‘very likely to be the cause’ and were more consistent with a case-level description by the same definition. Thus, only the 'case level' and 'clinical level' were utilised in this study (Table 3). The terms definitive (Category 1), possible (Category 2), candidate (Category 3) and negative (Category 4) were used based on the WES reports. The criteria used to determine the clinical-level classification included the phenotype-genotype correlation after discussion with experts; follow-up biochemical, radiological and functional studies; and additional molecular studies (e.g. X-inactivation). These criteria cannot be evaluated at the case-level-based on the initial laboratory report. The term 'unlikely' (Category 4) was used when a molecular diagnosis was deemed implausible after review (Table 3). The findings were considered concordant between case level and clinical level if the category was unchanged, e.g. definitive (Category 1). When a difference existed between the two, the classification was considered discordant and was indicated as promoted or demoted accordingly.

**Table 3:** Case-level classification and clinical-level classification

Categories	Case-level classification (Laboratory)	Case-level description	Clinical-level classification (Geneticist)	Clinical-level description
Category 1 (Definitive result)	Definitive	Pathogenic or variant(s) likely pathogenic in a known disease gene associated with the reported phenotype.	Definitive	Pathogenic or variant(s) likely pathogenic in a known disease gene associated with the reported phenotype, after post-exome phenotype review, biochemical, radiological or functional studies is in accordance with the molecular diagnosis.
Category 2 (Possible/probable diagnosis)	Possible	Variant(s) in a known disease gene possibly associated with the reported phenotype. This category includes novel variants in disease genes that overlap the phenotype provided for the proband.	Likely	Novel variant(s) in known disease genes determined to be likely associated with patient phenotype, but evidence is insufficient for a definitive diagnosis after post-exome analysis.
Category 3 (Variant of uncertain significance or novel candidate gene)	Candidate	Variant(s) predicted to be deleterious in a novel candidate gene that have not previously been implicated in human disease or any variant for which the published data is insufficient to support human disease association.	Candidate	Variant(s) in novel candidate genes insufficient to support human disease association despite extensive post-exome analysis.
Category 4 (Negative result)	Negative	No variants in genes associated with the reported phenotype identified	Unlikely	Candidate variants deemed implausible and excluded after post-exome analysis.
			Negative	No variants in genes associated with the reported phenotype identified despite post-exome review.

The ‘case-level’ and ‘clinical-level’ classifications according to Baldridge et al. The ‘case-level’ assertion was a synthesis of all the molecular data in a single subject by the laboratory only. The clinical-level assertion refers to a later stage where the geneticists reassess the classifications using criteria such as: the phenotype-genotype correlation, follow-up biochemical, radiological and functional studies, and additional molecular studies (e.g. X-inactivation). These cannot be determined at the ‘case-level’ from the initial laboratory report. The term ‘unlikely’ (Category 4) was used for molecular diagnosis deemed implausible after review

### Targeted parental segregation analysis of singleton exome results

For the variants identified in the singleton WES, a targeted parental segregation analysis was performed by Sanger sequencing. To avoid confusion and for an equal comparison of the singleton and trio cases, the case-level classification was considered only after the segregation analysis. Thus, variants confirmed by parental segregation alone were not considered a change in classification due to the geneticist’s input.

### Evaluation of the time required to analyse the exome reports

A retrospective evaluation of the time spent by the geneticist on the analysis was performed to illustrate the proportion of cases requiring an extended period of review. Cases requiring extensive discussion or follow-up were defined as those requiring well over three hours of work by the geneticist and his research team. This time included time spent on literature reviews, discussion among the research team, communication with experts and arranging specialised tests. Such discussions, electronic communication and test requests were documented and in total would have required well over three hours of the geneticist’s time. A cut-off of three hours was chosen because this cut-off was more clinically relevant and less likely to be affected by recall bias (Supplementary Table 1).

## Electronic supplementary material

Supplementary materials combined

## Electronic supplementary material

**Supplementary information** accompanies the paper on the *npj Genomic Medicine* website (10.1038/s41525-018-0056-5).
