# Supplementary material for: Exome sequencing for paediatric-onset diseases: impact of the extensive involvement of medical geneticists in the diagnostic odyssey
Source: NPJ Genom Med. 2018 Aug 6;3:19. doi: 10.1038/s41525-018-0056-5 (PMC6079040; doi:10.1038/s41525-018-0056-5)
Supplement: Supplementary file 1 — Supplementary materials combined [file 41525_2018_56_MOESM1_ESM.pdf]

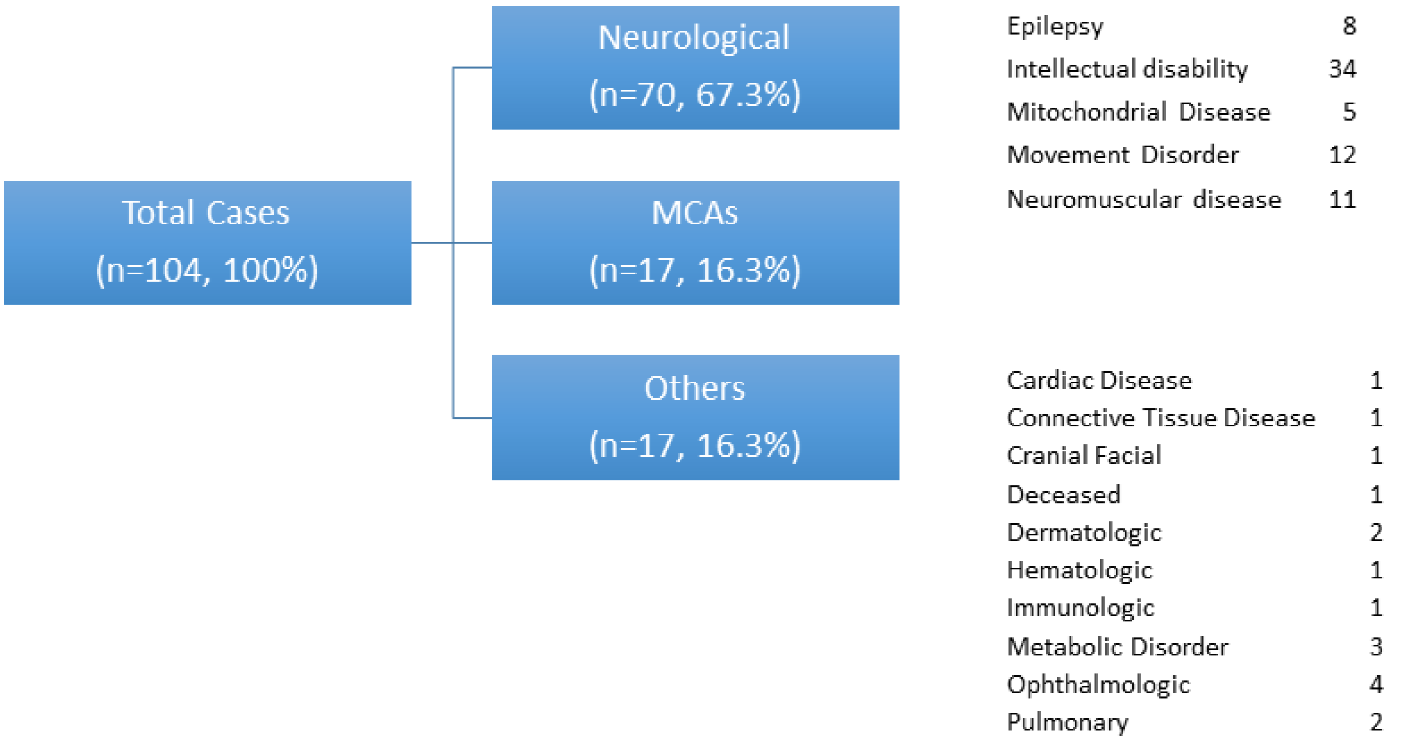

Supplementary Figure 1. Primary clinical indications for exome sequencing. The primary clinical indications were 1) neurological, 2) multiple congenital anomalies (MCAs) and 3) Others. Details of subcategories are listed.

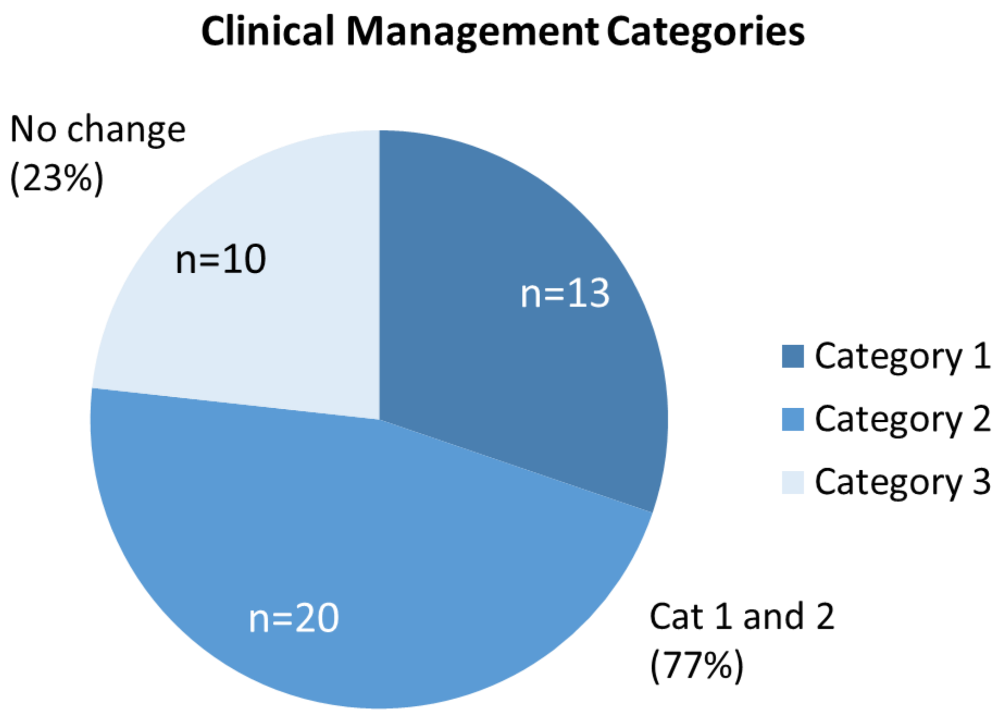

Clinical Management Categories	
Category 1	Disease-specific published management guidelines
Category 2	Management based on case reports or known function of genes
Category 3	No management change

Supplementary Figure 2. Management change categories in 43 conclusive diagnoses. Overall, 77% of positive diagnoses aided clinical management (Categories 1 and 2).

ID	Self-reported Chinese	Trios vs Singleton	Self-financed vs subsidized (Group)	Laboratory	Clinical categories	Subcategory	Confirmed Diagnosis (OMIM)
U001	Y	Trios	Group B	Ambry	Neurological	Intellectual disability	Mental retardation, X-linked 102 (OMIM #300958)
U002	N	Trios	Group B	Ambry	Other	Ophthalmologic	
U003	Y	Trios	Group B	Ambry	Neurological	Intellectual disability	Alpha-thalassemia/mental retardation syndrome, X-linked (OMIM #301040)
U004	Y	Trios	Group A	Ambry	Neurological	Movement Disorder	Epilepsy, focal, with speech disorder with or without mental retardation (OMIM #245570)
U005	Y	Trios	Group B	Ambry	Other	Hematologic	Fanconi anaemia, complementation group A (OMIM #227650)
U006	Y	Trios	Group B	Ambry	MCAs		Schuss-Hoeijmakers sydnrome (OMIM #615009)
U007	Y	Trios	Group B	Ambry	Other	Deceased	
U008	Y	Trios	Group B	Ambry	Other	Immunologic	
U009	Y	Singleton	Group A	Nijmegen	Neurological	Intellectual disability	
U010	Y	Singleton	Group A	Nijmegen	Other	Ophthalmologic	Axenfeld-Rieger syndrome, type 3 (OMIM #602482)
U011	Y	Trios	Group C	Nijmegen	MCAs		
U012	Y	Trios	Group C	Nijmegen	Neurological	Intellectual disability	10q26 microdeletion syndrome (OMIM #609623)
U013	Y	Singleton	Group B	Nijmegen	Neurological	Epilepsy	
U014	Y	Trios	Group B	Nijmegen	Other	Ophthalmologic	
U015	Y	Trios	Group B	Nijmegen	Neurological	Intellectual disability	Coffin-Siris syndrome 1 (OMIM #135900)
U016	Y	Singleton	Group B	Nijmegen	Neurological	Intellectual disability	
U017	Y	Trios	Group B	Nijmegen	Neurological	Movement Disorder	Autosomal recessive; Spastic paraplegia 11, autosomal recessive (OMIM #604360)
U018	Y	Singleton	Group A	Nijmegen	Neurological	Intellectual disability	
U019	Y	Singleton	Group A	Nijmegen	Neurological	Intellectual disability	
U020	Y	Singleton	Group A	Nijmegen	Neurological	Intellectual disability	
U021	Y	Singleton	Group A	Nijmegen	MCAs		
U022	Y	Singleton	Group A	Nijmegen	Neurological	Intellectual disability	
U023	Y	Singleton	Group A	Nijmegen	Neurological	Mitochondrial Disease	Beta-ueridopropionase deficiency (OMIM #613161)
U024	Y	Singleton	Group A	Nijmegen	Neurological	Intellectual disability	
U025	N	Singleton	Group A	Nijmegen	Neurological	Intellectual disability	Bainbridge-Ropers syndrome (OMIM# 615485)
U026	Y	Singleton	Group A	Nijmegen	MCAs		
U027	Y	Singleton	Group A	Nijmegen	MCAs		Shwachman-Bodian-Diamond syndrome (OMIM #260400)
U028	Y	Singleton	Group A	Nijmegen	Neurological	Mitochondrial Disease	Coenzyme Q10 deficiency, primary, 7 (OMIM #616276)
U029	Y	Singleton	Group A	Nijmegen	Neurological	Movement Disorder	
U030	Y	Trios	Group C	Nijmegen	Neurological	Intellectual disability	Coffin-Siris syndrome 2 (OMIM # 614607)
U031	Y	Singleton	Group A	Nijmegen	Neurological	Epilepsy	Hyperphosphatasia with mental retardation syndrome (HMRS) 2 (OMIM #614730)
U032	Y	Singleton	Group A	Nijmegen	Neurological	Intellectual disability	
U033	Y	Singleton	Group A	Nijmegen	MCAs		X-linked congenital disorder of glycosylation type Iim (OMIM #300896)

Supplementary Table 1. All 104 WES cases reported in this study. Group A: Singleton exome subsidized by research funding; Group B: Self-financed exome (singleton or trio); Group C: Trio exome (proband singleton exome subsidized, parental exomes self-financed)

U034	Y	Singleton	Group A	Nijmegen	Neurological	Mitochondrial Disease	
U035	Y	Singleton	Group A	Nijmegen	MCAs		
U036	Y	Singleton	Group A	Nijmegen	MCAs		
U037	Y	Singleton	Group A	Nijmegen	Neurological	Movement Disorder	
U038	Y	Singleton	Group A	Nijmegen	MCAs		
U039	Y	Singleton	Group A	Nijmegen	Neurological	Intellectual disability	Rubinstein-Taybi syndroom type 1 (OMIM #180849)
U040	Y	Singleton	Group A	Nijmegen	Neurological	Intellectual disability	Bainbridge-Ropers syndrome (OMIM# 615485)
U041	Y	Singleton	Group A	Nijmegen	Other	Metabolic Disorder	
U042	Y	Singleton	Group A	Nijmegen	Neurological	Movement Disorder	Early Infantile Epileptic Encephalopathy (OMIM #612164)
U043	Y	Singleton	Group A	Nijmegen	Neurological	Neuromuscular disease	
U044	Y	Singleton	Group A	Nijmegen	MCAs		Noonan syndrome-like disorder with loose anagen hair 2 (OMIM #617506)
U045	Y	Singleton	Group A	Nijmegen	MCAs		
U046	Y	Singleton	Group A	Nijmegen	Neurological	Intellectual disability	
U047	Y	Singleton	Group A	Nijmegen	MCAs		
U048	Y	Singleton	Group A	Nijmegen	Neurological	Movement Disorder	
U049	N	Singleton	Group A	Nijmegen	MCAs		Cutis laxa, autosomal recessive, type IID (OMIM #617403)
U050	Y	Singleton	Group A	Nijmegen	MCAs		Lenz-Majewski hyperostotic dwarfism (LMHD) (OMIM #151050)
U051	Y	Singleton	Group A	Nijmegen	Neurological	Neuromuscular disease	
U052	N	Singleton	Group A	Nijmegen	Neurological	Movement Disorder	Autosomal recessive agenesis of the corpus callosum with peripheral neuropathy (OMIM #218000)
U053	Y	Singleton	Group A	Nijmegen	Neurological	Intellectual disability	
U054	Y	Singleton	Group C	Nijmegen	Neurological	Neuromuscular disease	
U055	Y	Singleton	Group A	Nijmegen	Neurological	Movement Disorder	Wieacker-Wolff syndrome, X-linked recessive (OMIM #314580)
U056	Y	Singleton	Group A	Nijmegen	Neurological	Intellectual disability	
U057	N	Singleton	Group A	Nijmegen	Neurological	Epilepsy	
U058	Y	Singleton	Group A	Nijmegen	Neurological	Epilepsy	X-linked syndromic mental retardation, Turner Type (OMIM #300706)
U059	Y	Singleton	Group A	Nijmegen	MCAs		
U060	N	Singleton	Group A	Nijmegen	Other	Pulmonary	
U061	Y	Singleton	Group A	Nijmegen	Neurological	Epilepsy	
U062	Y	Trios	Group C	Nijmegen	Neurological	Intellectual disability	Neurodevelopmental disorder a.o. Rett syndrome (OMIM #312750)
U063	Y	Singleton	Group A	Nijmegen	Neurological	Neuromuscular disease	Congenital megaconial muscular dystrophy (OMIM #602541)
U064	Y	Singleton	Group A	Nijmegen	Other	Metabolic Disorder	
U065	Y	Singleton	Group A	Nijmegen	Neurological	Neuromuscular disease	
U066	Y	Singleton	Group A	Nijmegen	Neurological	Intellectual disability	
U067	Y	Singleton	Group A	Nijmegen	Neurological	Epilepsy	
U068	Y	Singleton	Group A	Nijmegen	Neurological	Intellectual disability	
U069	Y	Trios	Group C	Nijmegen	Neurological	Intellectual disability	Mental retardation, autosomal dominant 31 (OMIM #616158)

(continued) Supplementary Table 1.

U070	Y	Singleton	Group A	Nijmegen	Neurological	Neuromuscular disease	
U071	Y	Singleton	Group A	Nijmegen	Other	Connective Tissue Disease	
U072	Y	Singleton	Group C	Nijmegen	Neurological	Intellectual disability	
U073	Y	Singleton	Group A	Nijmegen	Neurological	Neuromuscular disease	
U074	Y	Singleton	Group A	Nijmegen	MCAs		Pallister-Killian syndrome (OMIM #601803)
U075	Y	Singleton	Group A	Nijmegen	Neurological	Intellectual disability	
U076	Y	Singleton	Group A	Nijmegen	Other	Dermatologic	Keratitis-ichthyosis-deafness syndrome (OMIM #148210)
U077	Y	Singleton	Group A	Nijmegen	Neurological	Intellectual disability	
U078	Y	Singleton	Group A	Nijmegen	Neurological	Epilepsy	
U079	Y	Singleton	Group A	Nijmegen	Other	Ophthalmologic	
U080	Y	Trios	Group C	Nijmegen	Neurological	Intellectual disability	Mental retardation, X-linked 102 (OMIM #300958)
U081	Y	Trios	Group C	Nijmegen	Neurological	Mitochondrial Disease	Desanto-Shinawi syndrome (OMIM #616708)
U082	Y	Singleton	Group A	Nijmegen	Other	Dermatologic	
U083	Y	Singleton	Group A	Nijmegen	Neurological	Intellectual disability	X-linked dominant Neurodegeneration with Brain Iron Accumulation 5, NBIA5 (OMIM #300894)
U084	Y	Singleton	Group A	Nijmegen	Neurological	Neuromuscular disease	Spinal muscular atrophy, lower extremity-predominant 1, AD (OMIM #158600)
U085	Y	Singleton	Group A	Nijmegen	Neurological	Intellectual disability	
U086	Y	Singleton	Group A	Nijmegen	Neurological	Intellectual disability	
U087	Y	Singleton	Group A	Nijmegen	Other	Cranial Facial	Auriculocondylar syndrome 1 (OMIM #602483)
U088	Y	Singleton	Group A	Nijmegen	Neurological	Intellectual disability	
U089	Y	Singleton	Group A	Nijmegen	Neurological	Neuromuscular disease	Insensitivity to pain, congenital, with anhidrosis (OMIM #256800)
U090	Y	Singleton	Group A	Nijmegen	Neurological	Mitochondrial Disease	Costello syndrome (OMIM #218040)
U091	Y	Singleton	Group A	Nijmegen	Other	Pulmonary	
U092	Y	Singleton	Group A	Nijmegen	Neurological	Neuromuscular disease	Myasthenic syndrome, congenital, 8, with pre- and postsynaptic defects (OMIM #615120)
U093	Y	Singleton	Group A	Nijmegen	Neurological	Movement Disorder	
U094	Y	Trios	Group A	Nijmegen	Other	Cardiac Disease	
U095	Y	Singleton	Group A	Nijmegen	Neurological	Epilepsy	
U096	Y	Singleton	Group A	Nijmegen	Other	Metabolic Disorder	
U097	Y	Trios	Group B	Nijmegen	Neurological	Movement Disorder	
U098	Y	Singleton	Group A	Nijmegen	Neurological	Neuromuscular disease	Charcot-Marie-Tooth disease, axonal, type 2S (OMIM #616155)
U099	Y	Singleton	Group A	Nijmegen	Neurological	Intellectual disability	Nicolaides-Baraitser syndrome (OMIM #601358)
U100	Y	Trios	Group B	Nijmegen	Neurological	Intellectual disability	Mental retardation, autosomal dominant 7 (OMIM #614104)
U101	Y	Singleton	Group A	Nijmegen	Neurological	Movement Disorder	
U102	Y	Trios	Group B	Ambry	Neurological	Movement Disorder	Dystonia 28, childhood-onset (OMIM #617284)
U103	Y	Trios	Group B	Nijmegen	MCAs		Aarskog-Scott syndrome (OMIM #305400)
U104	Y	Singleton	Group A	Nijmegen	Neurological	Intellectual disability	

(continued) Supplementary Table 1.

ID	Gene Panel Variant(s)	Exome Variant(s)
U001	Nil	DDX3X; NM_001356:c.1490C>T (p.(Ala497Val))
U002	Nil	negative
U003	Nil	ATRX; NM_000489:c.740A>G (p.(Asn247Ser))
U004	Nil	GRIN2A; NM_000833.4:c.1841A>G (p.(Asn614Ser))
U005	Nil	FANCA; NM_001286167.2:c.2941T>C (p.(Cys981Arg))
U006	Nil	PACS1; NM_018026.3:c.607 C>T (p.(Arg203Trp))
U007	Nil	negative
U008	Nil	negative
U009	negative	negative
U010	FOXC1; Chr6(GRCh37):g.1611072C>A; NM_001453.2:c.392C>A (p.(Ser131*); heterozygous	
U011	negative	negative
U012	negative	CNV -Monosomy 10q26.2-qter
U013	negative	negative
U014	negative	negative
U015	ARID1B; Chr6(GRCh37):g.157502101A>G; NM_020732.3:c.3136-2A>G (r.spl?) heterozygous	
U016	negative	negative
U017	negative	SPG11; Chr15(GRCh37):g.44905697dup; NM_025137.3:c.3075dup (p.(Glu1026fs)); heterozygous; paternal<br>SPG11; Chr15(GRCh37):g.44887457-?_44889141+?del; deletion of exon 24-26 (NM_025137.3) unknown breakpoints (HGVS: NM_025137.3:c.4002-?_4635+?del (p.?); heterozygous; maternal
U018	negative	negative
U019	negative	negative
U020	negative	negative
U021	negative	Negative
U022	negative	negative
U023	negative	UPB1; Chr22(GRCh37):g.24919647G>A; NM_016327.2: c.977G>A (p.Arg326Gln); homozygous
U024	MECP2; ChrX(GRCh37):g.153296153G>A; NM_004992.3:c.1126C>T (p.(Pro376Ser)) heterozygous	negative
U025	negative	ASXL3; NM_030632.2:c.4399C>T (p.(Arg1467Ter))
U026	FUCA1; Chr1(GRCh37):g.24192112A>T; NM_000147.4:c.393T>A (p.(Tyr131*))	MYH3; Chr17(GRCh37):g.10541472_10541473delinsAT; NM_002470.3:c.3616_3617delinsAT
U027	GRIN2A; Chr16(GRCh37):g.9858774A>G; NM_000833.3:c.2627T>C (p.(Ile876Thr)); heterozygous	SBDS; Chr7(GRCh37):g.66459273T>A; NM_016038.2:c.184A>T (p.(Lys62*)); heterozygous<br>SBDS; Chr7(GRCh37):g.66459197A>G; NM_016038.2:c.258+2T>C (r.spl?)); heterozygous<br>SBDS; Chr7(GRCh37):g.66459197A>G; NM_016038.2:c.258+2T>C (r.spl?)); heterozygous
U028	COQ4; Chr9(GRCh37):g.131088161G>A; NM_016035.3: c.402+1G>A (p.?); heterozygous<br>COQ4; Chr9(GRCh37):g.131095146T>C; NM_016035.3: c.550T>C (p.Trp184Arg); heterozygous	
U029	SPAST; Chr2(GRCh37):g.32341228T>G; NM_014946.3:c.1045T>G (p.(Leu349Val)); heterozygous<br>SPAST; Chr2(GRCh37):g.32379538C>G; NM_014946.3:c.1824C>G (p.(Asn608Lys)); heterozygous	negative
U030	ARID1A; Chr1(GRCh37):g.27097817G>A; NM_006015.4:c.3406G>A (p.(Ala1136Thr)) heterozygous	
U031	PIGO; Chr9(GRCh37):g.35091693; NM_032634.3:c.2191del (p.(Arg731fs)) heterozygous<br>PIGO; Chr9(GRCh37):g.35095105; NM_032634.3:c.458T>C (p.Phe153Ser)) heterozygous	
U032	negative	negative
U033	SLC35A2; ChrX(GRCh37):g.48762617dup; NM_005660.1:c.569dup (p.(Gly191fs)) heterozygous	

(continued) Supplementary Table 1.

U034	negative	negative
U035	negative	negative
U036	negative	MN1
U037	SPG7; Chr16(GRCh37):g.89614409_89614410del; NM_003119.3:c.1553-2_1553-1del (r.spl?) heterozygous	CAMTA1; Chr1(GrCh37):g.7723649C>G; NM_015215.3: c.1042C>G (p.(Gln348Glu)); heterozygous
U038	FLNA; ChrX(GRCh37):g.153588198T>G; NM_001110556.1:c.3881A>C (p.(Lys1294Thr)); heterozygous<br>RET; Chr10(GRCh37):g.43600607C>A;NM_020975.4:c.833C>A (p.(Thr278Asn)); heterozygous	negative
U039	CREBBP; Chr16(GRCh37):g.3789726C>A; NM_004380.2:c.4134-1G>T (r.spl?) heterozygous	NF1; Chr17(GRCh37):g29556972_29556974del; NM_001042492.2:c.2970_2972del; p.Met992del.
U040	ASXL3; Chr18(GRCh37):g.31318745_31318746del; NM_030632.1:c.1377_1378del (p.(Glu459fs));heterozygous	
U041	negative	negative
U042	negative	STXBP1; Chr9(GRCh37):g.130438134C>T; NM_003165.3:c.1162C>T (p.(Arg388*)) heterozygous
U043	MYH3;Chr17:10541426_10541428del; NM_002470.3:c.3661_3663del (p.(Glu1221del)) heterozygous	negative
U044	(not included)	negative
U045	negative	negative
U046	negative	negative
U047	negative	negative
U048	SCN8A; Chr12(GRCh37):g.52056654C>A; NM_014191.3:c.53C>A (p.(Thr18Asn)) heterozygous	negative
U049	ATP6V1A:NM_001690.3:c.215G>A<br>FAT4:NM_024582.4:c.11501C>T	
U050	PTDSS1; Chr8(GRCh37):g.97316346G>T; NM_014754.1:c.831G>T (p.(Trp277Cys)); heterozygous	
U051	COL6A2; Chr21(GRCh37):g.47540454G>A; NM_001849.3:c.1358G>A (p.(Arg453His)); heterozygous<br>COL6A2; Chr21(GRCh37):g.47544599G>A; NM_001849.3:c.1706G>A (p.(Arg569Gln)); heterozygous<br>COL6A2; Chr21(GRCh37):g.47552409C>A; NM_001849.3: c.3003C>A (p.(Asp1001Glu)); heterozygous	Exome negative
U052	negative	SLC12A6; NM_133647.1: c.3031C>T (p.(Arg1011*)); homozygous
U053	negative	negative
U054	negative	negative
U055	negative	ZC4H2; NM_018684.2: c.593G>A, p.Arg198Gln
U056	negative	negative
U057	negative	negative
U058	negative	HUWE1; ChrX(GRCh37):g.53631677C>T; NM_031407.6:c.2615G>A (p.(Arg872Gln)) hemizygous<br>CACNA1H; Chr16(GRCh37):g.1245967C>T; NM_021098.2:c.587C>T (p.(Ser196Leu)) heterozygous
U059	negative	negative
U060	negative	negative
U061	negative	FSCN2; NM_001077182.2:c.673G>T (p.(Asp225Tyr)) heterozygous
U062	MECP2; ChrX(GRCh37):g.153296777G>A; NM_001110792.1:c.538C>T (p.(Arg180*)) heterozygous, de novo	
U063	CHKB; NM_005198.4:c.598del (p.(Gln200fs)) homozygous	
U064	negative	negative
U065	negative	negative
U066	Negative	MFN2: Chr1(GRCh37):g.12058934C>T; NM_014874.3:c.707C>T (p.(Thr236Met)); heterozygous
U067	negative	negative
U068	CSNK2A1; Chr20(GRCh37):g.472926T>C; NM_177559.2:c.593A>G (p.(Lys198Arg)) heterozygous	
U069	PURA; Chr5(GRCh37):g.139494073_139494074del; NM_005859.4:c.307_308del (p.(Ser103fs)) heterozygous, de novo	not performed

(continued) Supplementary Table 1.

U070	Negative	GJB2; Chr13(GRCh37):g.20763612C>T; NM_004004.5:c.109G>A (p.(Val37Ile)) homozygous<br>RYR1; Chr19(GRCh37):g.38959977C>G; NM_000540.2:c.3589C>G (p.(Gln1197Glu)) heterozygous
U071	Negative	Negative
U072	negative	TGM6; NM_198994.2:c.1528G>C; p.510H
U073	negative	negative
U074	negative	CNV -dup(12)(pterp11.1)
U075	negative	negative
U076	NM_004004.5:c148G>A (p.(Asp50Asn)) heterozygous	not performed
U077	negative	negative
U078	negative	negative
U079	negative	negative
U080	DDX3X; ChrX(GRCh37):g.41205628del; NM_001356.4:c.1462del (p.(Arg488fs)); heterozygous, de novo	not performed
U081	negative	WAC; Chr10(GRCh37):g.28899633dupA; NM_016628.4: c.1171dupA (p.(Thr391Asnfs*15)); heterozygous; DE NOVO
U082	FLG: Chr1(GRCh37):g.152275298T>A; NM_002016.1:c.12064A>T; p.(Lys4022*); HETEROZYGOUS	
U083	WDR45; ChrX(GRCh37):g.48934399C>T; NM_007075.3:c.249G>A (p.(Trp83*)); heterozygous	
U084	DYNC1H1; Chr14(GRCh37):g.102446288C>T; NM_001376.4:c.751C>T (p.(Arg251Cys)) heterozygous<br>RYR1; Chr19(GRCh37):g.38954087C>T; NM_000540.2:c.2602C>T (p.(Arg868Cys)) heterozygous<br>BICD2; Chr9(GRCh37):g.95526894C>G; NM_001003800.1:c.133G>C (p.(Glu45Gln)) heterozygous	not performed
U085	negative	ZMYM3; ChrX(GRCh37):g.70465837G>A; NM_005096.3:c.2684C>T(p.(Pro895Leu))
U086	TAF1:c.2482C>G (p.(Leu828Val)); hemizygous	
U087	GNAI3; Chr1(GRCh37):g.110116380G>A; NM_006496.3:c.140G>A (p.(Ser47Asn)) heterozygous.	
U088	CDKL5: ChrX(GRCh37):g.18622260_18622262del; NM_001037343.1:c.1216_1218del (p.(Leu406del)) heterozygous	
U089	NTRK1:NM_002529.3:c.744del<br>NTRK1:NM_002529.3:c.2089G>A	
U090	HRAS; Chr11(GRCh37):g.533467C>G; NM_005343.2:c.436G>C (p.(Ala146Pro)); heterozygous	
U091	negative	negative
U092	AGRN; Chr1(GRCh37):g.983155G>A; NM_198576.3: c.3632-1G>A (r.spl?) homozygous	negative
U093	negative	OLIG1; Chr21(GRCh37):g.34442554T>G; NM_138983.2:c.2T>G (p.(Met1?)) heterozygous
U094	negative	FLNA; ChrX(GRCh37):g.153580001G>A; NM_001110556.1:c.6971C>T (p.(Pro2324Leu)); hemizygous<br>XPNPEP2; ChrX(GRCh37):g.128876157del; NM_003399.5:c.123del (p.(Tyr42Thrfs*37)); hemizygous
U095	negative	negative
U096	negative	ABCB11; Chr2(GRCh37):g.169783855G>C; NM_003742.2:c.3429C>G (p.(Asp1143Glu)); heterozygous<br>ABCB11; Chr2(GRCh37):g.169801131G>A; NM_003742.2:c.2594C>T (p.(Ala865Val)); heterozygous
U097	negative	negative
U098	IGHMBP2; Chr11(GRCh37):g.68704304del; NM_002180.2:c.2356del (p.(Ala786fs)) heterozygous<br>SYT2; Chr1(GRCh37):g.202569556G>C; NM_177402.4:c.848C>G (p.(Thr283Arg)) heterozygous	IGHMBP2; c.903_912+83del
U099	SMARCA2;Chr9(GRCh37):g.2110344C>A; NM_003070.4:c.3383C>A (p.(Ala1128Asp)) heterozygous, de novo<br>SMARCA2;Chr9(GRCh37):g.2110355G>C; NM_003070.4:c.3394G>C (p.(Gly1132Arg)) heterozygous, de novo	
U100	DYRK1A; Chr21(GRCh37):g.38877628G>T;NM_001396.3:c.1282G>T (p.(Gly428*)); heterozygous, de novo	
U101	negative	
U102	nil	KMT2B;NM_014727:c.2425C>T (p.(Q809*))
U103	negative	negative
U104	negative	

(continued) Supplementary Table 1.

ID	Laboratory Case level classification	After Segregation	Clinical geneticist clinical-level classification	>3Hrs to Review
U001	Negative	Negative	Definitive	
U002	Negative	Negative	Negative	
U003	Possible	Possible	Definitive	
U004	Definitive	Definitive	Definitive	
U005	Definitive	Definitive	Definitive	
U006	Definitive	Definitive	Definitive	
U007	Negative	Negative	Negative	
U008	Negative	Negative	Negative	
U009	Negative	Negative	Negative	
U010	Definitive	Definitive	Definitive	
U011	Negative	Negative	Negative	
U012	Negative	Negative	Definitive	
U013	Negative	Negative	Negative	
U014	Negative	Negative	Negative	
U015	Definitive	Definitive	Definitive	
U016	Negative	Negative	Negative	
U017	Definitive	Definitive	Definitive	
U018	Negative	Negative	Negative	
U019	Negative	Negative	Negative	
U020	Negative	Negative	Negative	
U021	Negative	Negative	Negative	
U022	Negative	Negative	Negative	
U023	Candidate	Candidate	Definitive	Yes
U024	Possible	Possible	Unlikely	Yes
U025	Negative	Negative	Definitive	Yes
U026	Possible	Unlikely	Unlikely	Yes
U027	Candidate	Candidate	Definitive	Yes
U028	Possible	Possible	Definitive	Yes
U029	Candidate	Candidate	Unlikely	Yes
U030	Candidate	Candidate	Definitive	
U031	Possible	Possible	Definitive	Yes
U032	Negative	Negative	Negative	
U033	Possible	Possible	Definitive	Yes

(continued) Supplementary Table 1.

U034	Negative	Negative	Negative	
U035	Negative	Negative	Negative	
U036	Candidate	Candidate	Candidate	Yes
U037	Candidate	Candidate	Candidate	Yes
U038	Negative	Negative	Negative	
U039	Possible	Possible	Definitive	Yes
U040	Possible	Definitive	Definitive	
U041	Negative	Negative	Negative	
U042	Definitive	Definitive	Definitive	
U043	Possible	Unlikely	Unlikely	Yes
U044	Negative	Negative	Definitive	Yes
U045	Negative	Negative	Negative	
U046	Negative	Negative	Negative	
U047	Negative	Negative	Negative	
U048	Candidate	Unlikely	Unlikely	
U049	Candidate	Candidate	Definitive	Yes
U050	Possible	Definitive	Definitive	
U051	Candidate	Candidate	Unlikely	Yes
U052	Definitive	Definitive	Definitive	
U053	Negative	Negative	Negative	
U054	Negative	Negative	Negative	
U055	Definitive	Definitive	Definitive	
U056	Negative	Negative	Negative	
U057	Negative	Negative	Negative	
U058	Candidate	Candidate	Likely	Yes
U059	Negative	Negative	Negative	
U060	Negative	Negative	Negative	
U061	Candidate	Unlikely	Unlikely	Yes
U062	Definitive	Definitive	Definitive	
U063	Definitive	Definitive	Definitive	
U064	Negative	Negative	Negative	
U065	Negative	Negative	Negative	
U066	Possible	Unlikely	Unlikely	
U067	Negative	Negative	Negative	
U068	Candidate	Candidate	Definitive	Yes
U069	Definitive	Definitive	Definitive	

(continued) Supplementary Table 1.

U070	Possible	Possible	Unlikely	Yes
U071	Negative	Negative	Negative	
U072	Negative	Negative	Negative	Yes
U073	Negative	Negative	Negative	
U074	Negative	Negative	Definitive	
U075	Negative	Negative	Negative	
U076	Definitive	Definitive	Definitive	Yes
U077	Negative	Negative	Negative	
U078	Negative	Negative	Negative	
U079	Negative	Negative	Negative	
U080	Definitive	Definitive	Definitive	
U081	Definitive	Definitive	Definitive	
U082	Candidate	Candidate	Candidate	Yes
U083	Candidate	Definitive	Definitive	
U084	Definitive	Definitive	Definitive	
U085	Candidate	Candidate	Candidate	
U086	Possible	Possible	Unlikely	Yes
U087	Definitive	Definitive	Definitive	
U088	Candidate	Unlikely	Unlikely	
U089	Definitive	Definitive	Definitive	
U090	Definitive	Definitive	Definitive	
U091	Negative	Negative	Negative	
U092	Definitive	Definitive	Definitive	
U093	Candidate	Candidate	Candidate	
U094	Candidate	Candidate	Candidate	Yes
U095	Negative	Negative	Negative	
U096	Possible	Possible	Unlikely	
U097	Negative	Negative	Negative	
U098	Candidate	Candidate	Definitive	Yes
U099	Possible	Definitive	Definitive	
U100	Definitive	Definitive	Definitive	
U101	Negative	Negative	Negative	
U102	Definitive	Definitive	Definitive	
U103	Negative	Negative	Definitive	Yes
U104	Negative	Negative	Negative	

(continued) Supplementary Table 1.

Management interventions	Description
R	referral to a specialist (at least one time) for management of potential complication(s) associated with genetic variant
D	diagnostic testing (e.g. lab tests, echo and other imaging) indicated to evaluate possible complications that would not otherwise have been suspected
P	surgical/interventional procedure may result or be contraindicated
S	surveillance (recurrent) for potential complication(s) associated with genetic variant (e.g. medical exam or diagnostic imaging)
M	medication to treat some aspect of the condition (either to consider prescribing or medication might be contraindicated)
L	lifestyle changes to mitigate some risk of the condition
O	other

Supplementary Table 2. Types of management interventions by Riggs et al.
Management implications for each positive diagnosis were categorized into one of seven categories, provided as supplementary details to Table 3.
